# Fatal Cases of Seasonal Influenza in Russia in 2015–2016

**DOI:** 10.1371/journal.pone.0165332

**Published:** 2016-10-24

**Authors:** T. Ilyicheva, A. Durymanov, I. Susloparov, N. Kolosova, N. Goncharova, S. Svyatchenko, O. Petrova, A. Bondar, V. Mikheev, A. Ryzhikov

**Affiliations:** 1 Vector State Research Center of Virology and Biotechnology, Koltsovo, Novosibirsk, Russia; 2 Novosibirsk State University, Novosibirsk, Russia; 3 Genomics Core Facility, Institute of Chemical Biology and Fundamental Medicine, Siberian Branch of the Russian Academy of Sciences, Novosibirsk, Russia; Icahn School of Medicine at Mount Sinai, UNITED STATES

## Abstract

The influenza epidemic in 2015–2016 in Russia is characterized by a sharp increase of influenza cases (beginning from the second week of 2016) with increased fatalities. Influenza was confirmed in 20 fatal cases registered among children (0–10 years), in 5 cases among pregnant women, and in 173 cases among elderly people (60 years and older). Two hundred and ninety nine people died from influenza were patients with some chronic problems. The overwhelming majority among the deceased (more than 98%) were not vaccinated against influenza. We isolated 109 influenza A(H1N1)pdm09 and one A(H3N2) virus strains from 501 autopsy material samples. The antigenic features of the strains were similar to the vaccine strains. A phylogenic analysis of hemagglutinin revealed that influenza A(H1N1)pdm09 virus strains belonged to 6B genetic group that had two main dominant subgroups during the 2015–2016 season. In Russia strains of the first group predominated. We registered an increased proportion of strains with D222G mutation in receptor-binding site. A herd immunity analysis carried out immediately prior to the epidemic showed that 34.4% blood sera samples collected in different regions of Russia were positive to A/California/07/09(H1N1)pdm09. We came to a conclusion that public awareness enhancement is necessary to reduce unreasonable refusals of vaccination.

## Introduction

According to the World Health Organization (WHO) Regional Office for Europe, the influenza epidemic season started in week 51, 2015. The beginning of the season in Eastern Europe and Western Asia was marked by a spike of influenza cases compared to Western and South Europe. Five countries (Armenia, Georgia, Russia, Serbia and Ukraine) reported a rising incidence of severe acute respiratory infections (SARI) cases [1].

Predominance of A(H1N1)pdm09 subtype among circulating influenza viruses was observed in all Northern hemisphere countries except China where cocirculation of A(H1N1)pdm09, A(H3N2) and influenza B was registered. Some European countries (Italy, Slovenia, Turkey and France) reported cases caused by influenza A(H3N2) viruses. According to FluNet, influenza B viruses cocirculated together with A(H1N1)pdm09 viruses in a number of countries.

In Russia a morbidity spate started in the week 2 of 2016; and the influenza epidemic season finished by the 12^th^ week. Influenza A(H1N1)pdm09 virus was predominant during the whole period of epidemic. Laboratory monitoring confirmed more than 28,200 influenza cases including approximately 28,000 influenza A cases, of which 93% were caused by A(H1N1) virus [2].

We have been studying the condition of herd immunity and influenza epidemical situation in Russia since 2009[3–5]. This paper focuses on the herd immunity to influenza immediately before the 2015–2016 epidemics in Russia and evaluates the confirmed fatal cases of influenza in that epidemic season.

## Materials and Methods

### Study of herd immunity

Blood samples were obtained on condition of anonymity upon written informed donor consents. Sera were collected at the Sanitary-and-Epidemiological Centers of the Federal Service for Supervision of Consumer Rights and Human Welfare in 34 regions of Russia, 100–200 pieces per region. The places of collection located close to flyways and breeding grounds of wild waterfowls. Sera were collected from healthy donors. 5-ml blood samples were collected using disposable syringes or disposable plastic systems (vacutainers). The samples were transported to Vector State Research Center of Virology and Biotechnology (SRC VB Vector), Novosibirsk, in insulated shipping containers with cold packs. The collected samples were stored at –20°C before examination. The presence of antibodies to different types/ serotypes of influenza virus in the sera was tested following a standard technique, in hemagglutination inhibition (HI) test [6]. Testing of blood sera was approved by the Ethics Committee IRB 00001360 affiliated with SRC VB Vector (No.2 d.d Protocol, May 20, 2008) (S1 and S2 Files).

A/California/07/09(H1N1)pdm09, A/Switzerland/9715293/13(H3N2), B/Brisbane/60/2008 (Victoria lineage), B/Phuket/3073/2013 (Yamagata lineage) influenza viruses were kindly provided by the WHO Collaborating Center in Atlanta, the United States. The WHO Collaborating Center in Beijing, China, kindly furnished A/Anhui/01/2013(H7N9) virus. A/rook/Chany/32/2015(HPAI H5N1) virus (clade 2.3.2.1c.) was isolated by the authors in 2015 in Western Siberia [7].

### Influenza virus isolation from autopsy material

Samples were collected at the local Sanitary-and-Epidemiological Centers of the Federal Service for Supervision of Consumer Rights and Human Welfare after getting written informed consents from close relatives in accordance with the regulations of the Russian Federation. PCR-based diagnostics of raw material for influenza virus RNA was conducted in local laboratories, and then all the positive samples were sent to SRC VB Vector. Work with autopsy materials was approved by the Ethics Committee IRB 00001360 affiliated with SRC VB Vector (No.2 d.d. Protocol, May 20, 2008) (S1 and S2 Files).

After delivery to SRC VB Vector we tested all samples in PCR again and isolated strains in MDCK-London by infecting a monolayer of cells. For the purpose, the autopsy material was homogenized, a 10% suspension was prepared by adding the MEM culture medium (by Invitrogen), the suspension was filtered through a bacterial filter (0.22 μm pore diameter), and 200 μl were added to the culture flasks with a one-daymonolayer of MDCK cells, then we added medium MEM (Invitrogen), TPCK treatedtrypsin 2 μg/ml (Sigma), BCA0.2% and an antibiotic (Anti-Anti, Gibco). The infected cells were incubated at 37°C. The viral replication was monitored visually for cytopathic effect in HI test with chicken, goose and guinea pig erythrocytes and with human erythrocytes of 0(I)Rh group as well as in real-time PCR. If necessary, each sample was passaged at least three times.

### Typing / subtyping of influenza viruses

Typing / subtyping of isolated influenza virus strains and studying their antigenic features were carried out in HI test by the technique recommended by WHO [6] using postinfectious ferret reference sera kindly furnished by the WHO Collaborating Center on Influenza in Atlanta, the United States. HI test results were confirmed by real-time PCR. For the purpose, we relied on a kit of reagents for detecting RNA of influenza A virus (Influenzavirus A) and influenza B virus (Influenzavirus В) using PCR with hybridization-fluorescence detection, AmpliSenseInfluenza virus A/B-FL manufactured by the Central Research Institute of Epidemiology, the Federal Service for Supervision of Consumer Rights and Human Welfare (Moscow, Russia).

### Sequence analysis of hemagglutinin, neuraminidase and NS genes

Sequencing was carried out at the Genomics Core Facility, the Institute of Chemical Biology and Fundamental Medicine, the Siberian Branch of the Russian Academy of Sciences, Novosibirsk. To determine a nucleotide sequence of viral genome, viral RNA was isolated using the SV Total RNA Isolation System (Promega Corporation, Madison) according to the manufacturer’s instruction. Reverse transcription reaction was carried out with Uni12 primers using AMV reverse transcriptase. The products of amplification were isolated using QIAquick gel extraction kit (QIAGEN, Valencia). DNA sequencing was performed at the Genomics Core Facility, Novosibirsk. An automatic sequenator 3130xl Genetic Analyzer (Applied Biosystems, Foster City) was employed to analyze the products. The nucleotide sequences were studied using Vector NTI Advance 10 program package (Invitrogen, Carlsbad) and entered into the GISAID database. The phylogenetic trees were constructed applying the neighbor-joining method with the Kimura 2-parameter model and 5.2 MEGA version (www.megasoftware.net) with 1,000 bootstrap replicates.

### Sensitivity to antineuraminidase drugs

Sensitivity to antineuraminidase drugs was tested with the fluorescent method according to the WHO-recommended protocol [8].

## Results

### Investigation of the herd immunity

We studied herd immunity to influenza prior to the epidemic season. In October–November 2015 we collected 3,969 blood sera samples. None of the samples produced positive results with A(H5N1) and A(H7N9) antigens even at 1:10 dilution.

Table 1 shows the HI test results with antigens of A/California/07/09(H1N1)pdm09, A/Switzerland/9715293/13(H3N2), B/Brisbane/60/2008 (Victoria lineage), B/Phuket/3073/2013 (Yamagata lineage) influenza viruses.

**Table 1:** Seropositivity among study groups.

Region of sample collection	Region 1 European Russia					Region 2 Ural and Western Siberia					Region 3 Eastern Siberia and Russian Far East					Across all regions	
Age group	0–17	18–45	46–64	65>	Amount / %	0–17	18–45	46–64	65>	Amount / %	0–17	18–45	46–64	65>	Amount / %	Amount	%
Total sera, including sera positive to virus:	2 0.2%	717 52.7%	545 40.1%	96 7.1%	1360	44 3.2%	809 59.5%	481 35.4%	25 1.8%	1359	22 1.8%	696 55.7%	421 33.7%	111 8.9%	1250	3969	100
A(H1N1)pdm09	2 0.2%	328 24.1%	240 17.6%	23 1.7%	593/43.6%	21 1.6%	260 19.1%	115 8.5%	8 0.6%	404/29.7%	10 0.8%	224 17.9%	116 9.3%	18 1.4%	368/29.4%	1365	34.4
A(H3N2)	0 0%	59 4.3%	44 3.2%	11 0.8%	114/8.4%	14 1%	79 5.8%	46 3.4%	0 0%	139/10.2%	0 0%	46 3.7%	17 1.4%	2 0.2%	65/5.2%	318	8.0
B (Victoria)	0 0%	155 11.4%	111 8.2%	18 1.3%	284/20.9%	9 0.7%	287 21.1%	120 8.8%	4 0.3%	420/30.9%	5 0.4%	210 16.8%	130 10.4%	33 2.6%	378/30.2%	1093	27.5
B (Yamagata)	1 0.1%	410 30.2%	244 17.9%	52 3.8%	707/52.0%	38 2.8%	398 29.3%	209 15.4%	12 0.9%	657/48.3%	17 1.4%	313 25%	188 15%	76 6.1%	594/47.5%	1958	49.3

Table 1 demonstrates that before the 2015–2016 epidemic season herd immunity to influenza varied slightly across Russian regions. For instance, a number of sera positive to A/California/07/09(H1N1pdm09) was a little higher in the European part of Russia (43.6%) than in the Urals and Western Siberia (29.7%) or in Eastern Siberia and Russian Far East (29.4%). Highly alarming data were detected on herd immunity to H3N2 virus: only 8% sera from all regions were positive to A/Switzerland/9715293/13. The figure for the samples from Eastern Siberia and Russian Far East was much lower—5.2%.

### Isolation and characterization of influenza viruses isolated from the autopsy material

From December 2015 to April 2016 SRC VB Vector received 501 autopsy material samples from patients presumably died from influenza. Seventy-one per cent of all sera were collected in the European part of Russia, 22 per cent in the Urals and Western Siberia, and 7 per cent in Eastern Siberia and Russian Far East. Since the share of population in these regions is 74.2, 21.6 and 4.2 per cent respectively, it is obvious that the mortality rate caused by influenza was similar in all Russian regions. The majority of deaths fell within the 31–59 age group. This situation can be attributed to the fact that children under the age of 3 stay home. Preschoolers and school students are vaccinated at schools and day-care centers. University and college students, as well as the elderly have an opportunity to get a free vaccination. In autumn 2015 13.3 million children and adolescents and 25.9 million adults (generally senior citizens) were immunized under the free vaccination program. In total 45.3 million people (31.3% of the population) were vaccinated [2]. Moreover, the over-31 population on the average suffers from more chronic problems compared to the young. That seems to explain why the 31–59 age group is the most vulnerable segment of the population. The relevant patient characteristics are given in Table 2.

**Table 2:** Characteristics of the patients presumably died from influenza in 2015–2016.

Parameters	Numbers	Percent
**Sex**		
M	260	52
F	233	46
n/a	8	2
**Region**		
1 –European Russia	353	71
2 –Ural and Western Siberia	111	22
3 –Eastern Siberia and Russian Far East	37	7
**Age group**		
0–2	11	2
3–10	9	2
11–18	0	0
19–30	23	5
31–45	136	27
46–59	147	29
60 and older	173	35
n/a	2	0
**Pregnant women**	5	1
**Chronic problems**		
Chronic obstructive pulmonary disease	16	3
Other respiratory diseases (asthma, chronic bronchitis, etc.)	9	2
Cardiovascular system diseases	27	6
Diabetes	39	8
Oncological diseases	11	2
Obesity	65	13
Infectious diseases (acquired immune deficiency syndrome, hepatitis, tuberculosis)	9	2
Other	7	1
**Vaccinated against influenza in 2014–2015**	8	2
n/a	222	45
**Hospitalized**	467	93
**Total**	**501**	**100**

PCR analysis showed that samples from 497 deceased were positive to influenza A(H1N1)pdm09 RNA; 4 samples were positive to influenza A(H3N2) RNA.

We isolated 109 influenza A(H1N1)pdm09 and one influenza A(N3N2) virus strains from autopsy material from 501 died patients.

All 109influenza A(H1N1)pdm09 virus strains were characterized in HI test with ferret sera anti-A/California/07/2009. Only the HI titer of A/Kaluga/13/2016 isolated from patient immunized with influenza vaccine on October 30, 2015 was 4-fold lower than the vaccine strain. Fifteen strains were additionally studied in HI test with serum of 38-year-old woman immunized on October 28, 2015 with Grippol trivalent vaccine (ser. 300815). The titer was below 20 before vaccination and reached 640 one month after immunization with A/California/07/2009 virus. According to the antigenic characteristics, all studied strains were similar to A/California/07/2009, which was recommended by WHO for developing the vaccine for the current epidemic season. Table 3 shows the results for fifteen A(H1N1)pdm09 virus strains.

**Table 3:** Properties of influenza viruses isolated from autopsy material in 2015–2016 epidemic season.

Region of sampling*	Virus	Date of sample collection	Sex	Age	Date of vaccination	Passage (MDCK)	HI titer with ferret anti- A/California/07/2009	HI titer with human serum after vaccination with “Grippol”	The drug concentration required to reduce NA activity by 50%, IC50, nМ		Sequences published in GISAID		
									Oseltamivir	Zanamivir	HA	NA	NS
**Reference antigen**													
A/California/07/2009 (CDC, Atlanta, the United States)							2560	640	0.29	0.47			
Influenza antigen A/California/7/ 2009 (NIBSC, the United Kingdom)							2560	-	-	-			
XW (Oseltamivir resistant) [8]									84.2–280.6	0.2–0.8			
OSAKA (Zanamivir resistant) [8]									0.1–0.5	123.7–334.5			
**А(H1N1)pdm09isolated virus**													
1	A/Alania/03/2016	06.01.2016	f	4	no	1	2560	160	0.50	0.63	EPI694640	EPI694641	EPI694642
1	A/Stavropol/07/2016	07.01.2016	f	53	no	1	2560	160	0.21	0.75	EPI704203	EPI704204	EPI704205
1	A/Stavropol/11/2016	07.01.2016	f	27	no	1	2560	160	0.52	0.72	EPI704030	EPI704031	EPI704032
1	A/Kaluga/13/2016	01.02.2016	m	66	30.10.2015	1	640	80	0.20	0.37	EPI772567	EPI772568	EPI772569
1	A/Saint-Petersburg/10/2016	03.01.2016	m	36	no	1	2560	160	0.51	0.70	EPI700033	EPI700034	EPI700035
1	A/Saint-Petersburg/22/2016	08.01.2016	m	41	no	1	2560	160	0.75	0.90	EPI711130	EPI748416	EPI711131
1	A/Saint-Petersburg/31/2016	01.01.2016	m	46	no	1	2560	320	0.37	0.55	EPI711128	EPI749107	EPI711129
1	A/Saint-Petersburg/39/2016	17.01.2016	m	41	no	1	2560	320	0.72	1.10	EPI711208	EPI749108	EPI711209
1	A/Saint-Petersburg/41/2016	18.01.2016	m	61	no	1	1280	320	0.88	1.03	EPI711210	EPI749109	EPI711211
1	A/Saint-Petersburg/46/2016	17.01.2016	m	76	no	1	2560	160	0.56	0.76	EPI721829	EPI721830	EPI721831
1	A/Saint-Petersburg/50/2016	25.01.2016	m	45	no	1	1280	160	1.06	1.23	EPI748389	EPI748390	EPI748391
2	A/Orenburg/05/2016	09.01.2016	m	57	no	1	2560	320	0.41	0.50	EPI704206	EPI704207	EPI704208
2	A/Noyabrsk/3/2016	11.02.2016	f	55	no	1	1280	160	0.24	0.47	EPI772605	EPI772606	EPI772608
2	A/Novosibirsk/181/2016	13.02.2016	m	59	no	1	1280	160	0.62	0.75	EPI772612	EPI772613	EPI772614
3	A/Chita/317/2016	28.01.2016	f	48	no	1	1280	160	0.68	0.80	EPI772586	EPI772587	EPI772588
**А(H3N2)isolated virus**													
Region	A(H3N2) virus	Sampling	Sex	Age	Vaccination	Passage	HI titer with A/Switzerland/9715293/2013	HI titer with A/HongKong/4801/2014	Oseltamivir	Zanamivir	HA	NA	NS
**Reference antigen**													
A/Switzerland/9715293/13(H3N2)							1280	80	0.62	0.75			
A/HongKong/4801/2014 (H3N2)							80	640	0.78	0.93			
1	A/Saratov/5/2016	11.01.2016	m	61	no	2	80	640	0.67	0.40	EPI687295	EPI687829	EPI693968

* Regions of sampling: 1 –European Russia, 2 –Urals and Western Siberia, 3 –Eastern Siberia and Russian Far East.

The antigenic properties of A/Saratov/5/2016 (H3N2) isolated strain are similar to those of A/HongKong/4801/2014 (H3N2) strain with a 16-fold reduction of HI titer compared to A/Switzerland/9715293/13 strain (Table 3). 107 investigated viruses were sensitive to antineuraminidase drugs—oseltamivir and zanamivir, 2 of the strains had 50–100-fold reduced sensitivity to oseltamivir. We intend to publish the study results on the strains with reduced sensitivity to antineuraminidase in our next paper.

### Genetic analysis

**Fig 1:**
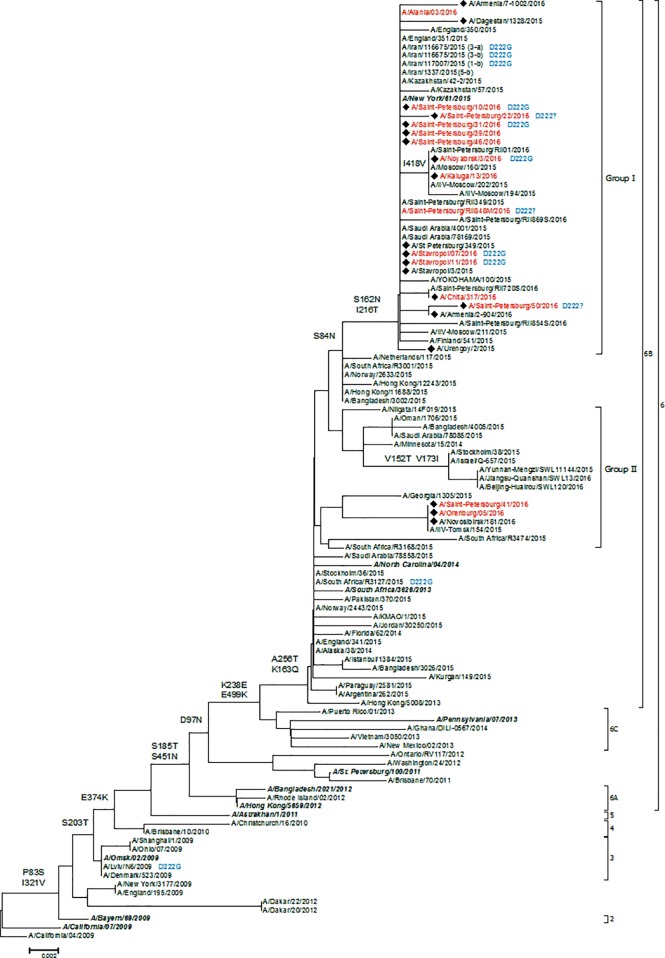
The phylogenetic tree of hemagglutinin protein of influenza A(H1N1)pdm09 virus. Bold italics–reference strains; red–strains obtained from confirmed fatal cases; boxed–genetic subgroups. On the tree topology the main characteristic mutations and individual mutations are highlighted. Black diamond (◆)–strains isolated by the authors.

**Fig 2:**
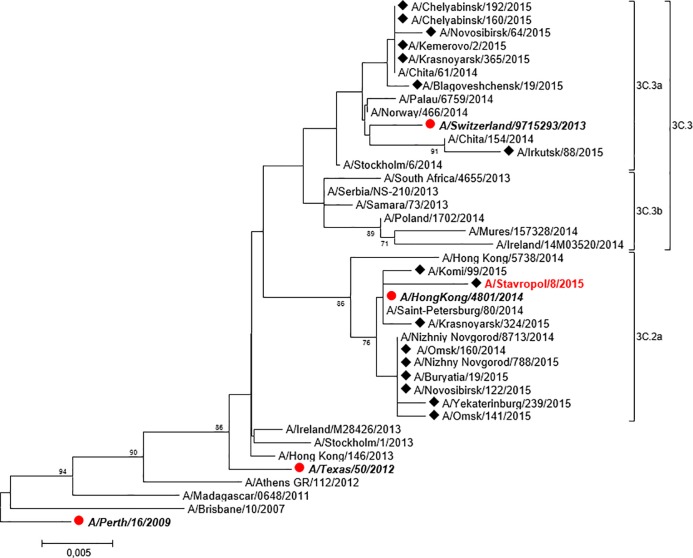
The phylogenetic tree of hemagglutinin protein of influenza A(H3N2) virus. Bold italics–vaccine strains; red–strain obtained from confirmed fatal cases in 2016; boxed–genetic subgroups. On the tree topology the main characteristic mutations and individual mutations are highlighted. Black diamond (◆)–strains obtained from confirmed fatal cases isolated by the authors.

We sequenced hemagglutinin (HA), neuraminidase (NA) and NS genes of 15 isolated A(H1N1)pdm09 viruses and 1 A(H3N2) virus (Table 3) and built up a phylogenetic tree of A(H1N1)pdm09 (Fig 1) and A(H3N2) HA (Fig 2).

HA phylogenic analysis revealed that influenza A(H1N1)pdm09 virus strains belonged to the 6B genetic group that had two main dominant subgroups during 2015–2016. In Russia strains of the first subgroup dominated. Among fatal cases we registered an increase in proportion of strains with D222G mutation in the receptor-binding site.

In all strains with a detected nucleotide sequence of NS gene we registered the following mutations: NS1 –N205S and NS2(NEP)–T48A which increase virulence [9] due to inhibition of IFN-α/β-dependent mechanism of the antiviral response in the infected cells (INF inhibition in culture).

Furthermore, we detected an NA gene mutation in one of the analyzed strains (A/Saint-Petersburg/41/2016) –S247N reducing sensitivity of this strain to NA inhibitor oseltamivir [10]. Nevertheless, our records indicate that this strain has standard sensitivity to oseltamivir.

We found no changes in A/Saratov/5/2016 (H3N2) NA resulting in resistance to antineuraminidase drugs, and in NS that might enhance pathogenicity. HA of this strain belongs to the same 3c.2a genetic group as A/HongKong/4801/2014 (H3N2) vaccine strain (Fig 2).

## Discussion

A(H1N1)pdm09 viruses have been circulating in the human community since 2009 and nine clades have been designated on HA phylogenetic tree. According to the European Centre for Disease Prevention and Control, “viruses in clade6, represented by A/St Petersburg/27/2011 and carrying amino acid substitutions of D97N, S185T and S203T in HA1 and E47K and S124N in HA2 compared with A/California/7/2009, have predominated worldwide with a number of subclades emerging. All viruses having characterized since September 2014 carry HA genes in 6B subclade, which is marked by additional amino acid substitutions of K163Q, A256T and K283E in HA1 and E172K in HA2 compared with A/California/7/2009, e.g. A/South Africa/3626/2013. A number of virus clusters have emerged within 6B clade and two of these have been designated as subclades: viruses in 6B.1 subclade are defined by HA1 amino acid substitutions S84N, S162N (which results in the formation of a new potential glycosylation motif at residues 162–164 of HA1) and I216T, while those in 6B.2 subclade are defined by HA1amino acid substitutions V152T and V173I” [11].

The European Centre for Disease Prevention and Control also states that “influenza A(H3N2) viruses circulated in 2015–2016 were poorly detected by reference-antisera to A/Switzerland/9715293/2013, i.e. vaccine strain recommended for usage in the Northern hemisphere in the 2015–2016 influenza season, although some viruses belonged to the same 3C.3a subclade as vaccine virus. Antisera to A/Hong Kong/4801/2014 virus recommended for developing influenza vaccines in the Southern hemisphere in 2016 and in the Northern hemisphere in 2016–2017 resulted in better detection of the analyzed viruses” [11].

In 2015–2016 in Russia fatal influenza cases were primarily caused by A(H1N1)pdm09 virus, and in 99% of confirmed fatal influenza cases we detected RNA of that virus subtype. Interestingly, the antigenic features of the strains were similar to A/California/7/2009 being recommended by WHO for vaccines since 2009, i.e. in the last seven epidemic seasons. A number of researchers believe that the increased mortality rate from influenza A(H1N1)pdm09 is caused by D222G mutation in HA protein [12–14]. Hemagglutinin D239F is also referred to as D222G or D225G in the literature with alternative (e.g. seasonal H1/H3) numberings. Emergence of the mutation leads to the alteration of virus binding to cell receptors: from human alpha-2,6 to avian-like alpha-2,3. The latter are more common in ciliated human cells of the lower respiratory airways [15, 16].

Some researchers state that this mutation is developed through selection from polymorphous influenza viruses. This selection is found in infected organisms, and at the stage of introduction of infection D222G mutation is neither present nor predominant [17]. Regenerating pneumocytes of the 2nd type comprise a type of sialic acids that provides predominant receptor binding between virions and D222G mutation. However, tropism of such mutant strains to the cells of human upper respiratory airways is reduced; thus, those strains are likely to have a reduced transmissibility among people. A high rate of regenerating pneumocytes of the 2nd type can be caused by chronic obstructive pulmonary disease. Climate or social-and-cultural characteristics of a particular region such as dusty air (dust storms, coal mines, smog), smoking, open-fire cooking cause formation of finely dispersed aerosols of non-soluble particles penetrating in lower airways enhancing regeneration of human lung cells [18], which can factor selection of influenza virus strains with an increased pathogenicity in the infected organisms. It is likely to be one of the feasible ways of selecting D222G mutation.

E391K mutation in HA gene (known as the 47th according to HA2 numeration) with capability to enhance virus pathogenicity has become typical for A(H1N1)pdm09 current strains [19]. This mutation is known to decrease the pH threshold value from 5.4 to 5.0 of HA conformation alteration leading to increased transmissibility and infectivity of a strain [20].

In the 2015–2016 season, two new yet uncharacterized mutations D2E and E125D (GISAID) were detected in a segment of nonstructural protein (NS) of influenza virus. E125D mutation is located at the site of binding to cell proteins causing production of antiviral mRNA. Such mutation seems to block antiviral cell activity.

Current strains include two more significant mutations in the segment of nonstructural protein in influenza viruses: T48A in NS2 and N205S in NS1 involved in enhancing antagonism to interferon alpha, which increases virulence [9]. This pair of mutations initially were globally registered in the NS segment of influenza A(H1N1)pdm09 viruses in the 2011–2012 season in India. Since the 2012–2013 season these mutations have been detected in the majority of influenza A(H1N1)pdm09 viruses isolated worldwide including Russia (according to GISAID).

Low herd immunity to A(H1N1)pdm virus can be another important factor increasing the number of more severe and fatal influenza cases. After emergence and prevalence of influenza A(H1N1)pdm09 virus variant in 2009–2011 this subtype has not predominated in the subsequent epidemic seasons [4] except the 2013–2014 season [3].

Consequently, new properties of influenza A(H1N1)pdm09 virus and low herd immunity in the recent years have resulted in an increased number of severe and fatal cases in Russia in the 2015–2016 influenza epidemic. Thus, there were 25 fatal cases caused by influenza A(H1N1)pdm09 [3] in the 2013–2014 season; three fatal cases in 2014–2015, and 497 fatal influenza cases in the 2015–2016 season. It is possible that the environmental conditions as well as social-and-cultural characteristics of the studied regions promoted selection of viruses with D222G mutation in HA involved in development of virus virulent properties in infected organisms.

In Russia immunization against seasonal influenza is on the national vaccination schedule. The following population categories should be vaccinated: 6-month old babies, schoolchildren and university students; transport, healthcare and education workers, pregnant women, elderly people at the age of 60 and older, as well as patients with chronic problems. However, in many cases people refuse vaccination for no important reasons. Undoubtedly, awareness-raising activities among the population should be intensified to decrease the number of unreasonable refusals of vaccination. For instance, in the 2015–2016 season in Russia 20 children aged 0–10, 5 pregnant women, and 173 elderly people at the age of 60 and older died from influenza; and 299 deceased persons had chronic illness. The majority of those who died (> 98%) were unvaccinated against influenza.

## Supporting Information

S1 File Conclusion of Ethical Committee 2008 in Russian. (PDF)

S2 File Conclusion of Ethical Committee 2008 in English. (DOCX)

## References

[1] Tjon-Kon-Fat R, Meerhoff T, Nikisins S, Pires J, Pereyaslov D, Gross D, et al The potential risks and impact of the start of the 2015–2016 influenza season in the WHO European Region: a rapid risk assessment. Influ Other Respir Viruses. 2016 10.1111/irv.12381 26918771 PMC4910174

[2] [Of the end of influenza and ARVI epidemic season in 2015–2016] Moscow: Federal Service for Supervision of Consumer Rights Protection and Human Welfare; 2016. Available: http://rospotrebnadzor.ru/about/info/news/news_details.php?ELEMENT_ID=5995&sphrase_id=638303. Accessed 28 May 2016.

[3] Ilyicheva T, Abdurashitov M, Durymanov A, Susloparov I, Goncharova N, Kolosova N, et al Herd immunity and fatal cases of influenza among the population exposed to poultry and wild birds in Russian Asia in 2013–2014. Journal of Medical Virology. 2016;88(1):35–44. 10.1002/jmv.24301 26105790

[4] Ilyicheva T, Sobolev I, Susloparov I, Kurskaya O, Durymanov A, Sharshov K, et al Monitoring of influenza viruses in Western Siberia in 2008–2012. Infection, Genetics and Evolution. 2013;20:117–87. 10.1016/j.meegid.2013.08.025 24012948

[5] Ilyicheva T, Susloparov I, Durymanov A, Romanovskaya A, Sharshov K, Kurskaya O, et al Influenza A/H1N1pdm virus in Russian Asia in 2009–2010. Infection, Genetics and Evolution. 2011;11(8):2107–12. 10.1016/j.meegid.2011.05.002 21600305

[6] Manual for the laboratory diagnosis and virological surveillance of influenza Geneva: WHO Global Influenza Surveillance Network; 2011. 153 p.

[7] Marchenko VY, Susloparov IM, Kolosova NP, Goncharova NI, Shipovalov AV, Ilyicheva TN, et al Highly pathogenic influenza H5N1 virus of clade 2.3.2.1c in Western Siberia. Archives of Virology. 2016:1–5. 10.1007/s00705-016-2800-4 26935914

[8] Hurt A. Fluorometric Neuraminidase Inhibition Assay Australia: WHO collaborating centre for reference & research on influenza; 2009. 10 p.

[9] Imai H, Shinya K, Takano R, Kiso M, Muramoto Y, Sakabe S, et al The HA and NS genes of human H5N1 influenza a virus contribute to high virulence in ferrets. PLOS Pathogens. 2010;6(9). 10.1371/journal.ppat.1001106 20862325 PMC2940759

[10] Boltz DA, Douangngeun B, Phommachanh P, Sinthasak S, Mondry R, Obert C, et al Emergence of H5N1 avian influenza viruses with reduced sensitivity to neuraminidase inhibitors and novel reassortants in Lao People's Democratic Republic. Journal of General Virology. 2010;91(4):949–59. 10.1099/vir.0.017459-0 20016036 PMC2888158

[11] Influenza virus characterisation, summary Europe, March 2016. Stockholm: European Centre for Disease Prevention and Control; 2016. 17 p.

[12] Lvov DK, Yashkulov KB, Prilipov AG, Burtseva EI, Shchelkanov MY, Shlyapnikova OV, et al Detection of amino acid substitutions of asparaginic acid for glycine and asparagine at the receptor-binding site of hemagglutinin in the variants of pandemic influenza A/H1N1 virus from patients with fatal outcome and moderate form of the disease. Voprosy Virusologii. 2010;55(3):15–8. 20608076

[13] Goka EA, Vallely PJ, Mutton KJ, Klapper PE. Mutations associated with severity of the pandemic influenza A(H1N1)pdm09 in humans: a systematic review and meta-analysis of epidemiological evidence. Archives of Virology. 2014;159(12):3167–83. 10.1007/s00705-014-2179-z 25078388

[14] Nguyen HKL, Nguyen PTK, Nguyen TC, Hoang PVM, Le TT, Vuong CD, et al Virological characterization of influenza H1N1pdm09 in Vietnam, 2010–2013. Influ Other Respir Viruses. 2015;9(4):216–24. 10.1111/irv.12323 25966032 PMC4474498

[15] Chutinimitkul S, Herfst S, Steel J, Lowen AC, Ye J, Van Riel D, et al Virulence-associated substitution D222G in the hemagglutinin of 2009 pandemic influenza A(H1N1) virus affects receptor binding. Journal of Virology. 2010;84(22):11802–13. 10.1128/JVI.01136-10 20844044 PMC2977876

[16] Casalegno J-S, Ferraris O, Escuret V, Bouscambert M, Bergeron C, Linès L, et al Functional Balance between the Hemagglutinin and Neuraminidase of Influenza A(H1N1)pdm09 HA D222 Variants. PLOS ONE. 2014;9(8):e104009 10.1371/journal.pone.0104009 25119465 PMC4131921

[17] Memoli MJ, Bristol T, Proudfoot KE, Sally Davis A, Dunham EJ, Taubenberger JK. In vivo evaluation of pathogenicity and transmissibility of influenza A(H1N1)pdm09 hemagglutinin receptor binding domain 222 intrahost variants isolated from a single immunocompromised patient. Virology. 2012;428(1):21–9. 10.1016/j.virol.2012.02.018 22575875 PMC3350642

[18] Kubysheva N, Soodaeva S, Postnikova L, Novikov V, Maksimova A, Chuchalin A. Associations between indicators of nitrosative stress and levels of soluble HLA-I, CD95 molecules in patients with COPD. COPD: Journal of Chronic Obstructive Pulmonary Disease. 2014;11(6):639–44. 10.3109/15412555.2014.898042 24884928

[19] Radomski JP, Płoński P, Zagórski-Ostoja W. The hemagglutinin mutation E391K of pandemic 2009 influenza revisited. Molecular Phylogenetics and Evolution. 2014;70(1):29–36. 10.1016/j.ympev.2013.08.020 24012880

[20] Maurer-Stroh S, Lee RTC, Eisenhaber F, Cui L, Phuah SP, Lin RTP. A new common mutation in the hemagglutinin of the 2009 (H1N1) influenza A virus. PLOS Currents. 2010;(6). 10.1371/currents.RRN1162 20535229 PMC2880458

