# Supplementary material for: Fatal Cases of Seasonal Influenza in Russia in 2015–2016
Source: PLoS One. 2016 Oct 24;11(10):e0165332. doi: 10.1371/journal.pone.0165332 (PMC5077104; doi:10.1371/journal.pone.0165332)
Supplement: S1 File — (PDF) [file pone.0165332.s001.pdf]

Федеральное бюджетное учреждение науки Государственный научный центр вирусологии и биотехнологии «Вектор»

ФБУН ГНЦ ВБ «ВЕКТОР»

Адрес: 630559 р.п.Кольцово Новосибирского района Новосибирской области
Телефон: (383) 336-60-10 Факс: (383) 336-74-09 E-mail: vector@vector.nsc.ru http://www.vector.nsc.ru
ОГРН 10554750448122 ИНН 5433161342

Решение Этического Комитета

ВЫПИСКА из протокола № 2 д.д. заседания Этического комитета при ФБУН ГНЦ ВБ «Вектор»

от «_20_» _мая_ 2008 г.

СЛУШАЛИ: докладчика и исполнителя проекта исследования А.М. Шестопалова (зав. отделом ГНЦ ВБ «Вектор») «Мониторинг за вирусом гриппа А у населения районов Сибири, неблагополучных по гриппу H5N1».

Рецензент проекта – д-р биол. наук, в.н.с. отдела молекулярной вирусологии флавивирусов и вирусных гепатитов И.А. Разумов

ПОСТАНОВИЛИ: Этический комитет при ГНЦ ВБ «Вектор» единогласно одобряет проект исследований «Мониторинг за вирусом гриппа А у населения районов Сибири, неблагополучных по гриппу H5N1» (руководящая организация ГНЦ ВБ «Вектор», исполнитель проекта – Шестопалов А.М., заведующий отделом зоонозных инфекций и гриппа ГНЦ ВБ «Вектор» и утверждает предложенный для рассмотрения пакет документов с учётом внесенных замечаний.

Председатель Этического комитета, канд. мед. наук — Ар.А. Сергеев

Секретарь ЭК — Ю. В. Кононова
