# Supplementary material for: Fatal Cases of Seasonal Influenza in Russia in 2015–2016
Source: PLoS One. 2016 Oct 24;11(10):e0165332. doi: 10.1371/journal.pone.0165332 (PMC5077104; doi:10.1371/journal.pone.0165332)
Supplement: S2 File — (DOCX) [file pone.0165332.s002.docx]

Federal Budgetary Research Institution “State Research Center
of Virology and Biotechnology Vector”

FBRI SRC VB “Vector"

Address: Koltsovo, Novosibirsk region, Russia, 630559

Phone: +7(383)336-60-10 Fax: +7(383)336-74-09

E-mail: vector@vector.nsc.ru http://www.vector.nsc.ru

Primary State Registration Number: 1055475044812

Taxpayer Identification Number: 5433161342

**Conclusion of Ethical Committee**

Extract from the protocol #2 d.d. of the meeting of Ethical Committee affiliated with FBRI SRC VB “Vector".

dated 20 May, 2008

**Heard:** A.M. Shestopalov (head of a department at FBRI SCR VB “Vector”), speaker and performer of the research project “Monitoring of influenza A virus in population from Siberia regions with adverse situation regarding influenza H5N1”

Project referee – I.A. Razumov, Dr. of Biological Sciences, leading researcher at Department of molecular virology of flaviviruses and viral hepatitis at FBRI SCR VB “Vector”.

**Resolved:** Ethical Committee affiliated with FBRI SCR VB “Vector” unanimously approved the research project “Monitoring of influenza A virus in population from Siberia regions with adverse situation regarding influenza H5N1” (principal organization – FBRI SCR VB “Vector”, project performer – A.M. Shestopalov, head of Department of zoonotic infections and influenza at FBRI SCR VB “Vector”) and approved a set of documents with amended corrections offered for consideration.

Chairman of Ethical Committee

Candidate of Medical Sciences Ar.A. Sergeev

Secretary of Ethical Committee Yu.V. Kononova
